# Interfering with Autophagy: The Opposing Strategies Deployed by *Legionella pneumophila* and *Coxiella burnetii* Effector Proteins

**DOI:** 10.3389/fcimb.2020.599762

**Published:** 2020-11-05

**Authors:** David R. Thomas, Patrice Newton, Nicole Lau, Hayley J. Newton

**Affiliations:** Department of Microbiology and Immunology, Peter Doherty Institute for Infection and Immunity, The University of Melbourne, Melbourne, VIC, Australia

**Keywords:** autophagy, type 4 secretion system (T4SS), effector protein, *Coxiella burnetii*, *Legionella pneumophila*

## Abstract

Autophagy is a fundamental and highly conserved eukaryotic process, responsible for maintaining cellular homeostasis and releasing nutrients during times of starvation. An increasingly important function of autophagy is its role in the cell autonomous immune response; a process known as xenophagy. Intracellular pathogens are engulfed by autophagosomes and targeted to lysosomes to eliminate the threat to the host cell. To counteract this, many intracellular bacterial pathogens have developed unique approaches to overcome, evade, or co-opt host autophagy to facilitate a successful infection. The intracellular bacteria *Legionella pneumophila* and *Coxiella burnetii* are able to avoid destruction by the cell, causing Legionnaires’ disease and Q fever, respectively. Despite being related and employing homologous Dot/Icm type 4 secretion systems (T4SS) to translocate effector proteins into the host cell, these pathogens have developed their own unique intracellular niches. *L. pneumophila* evades the host endocytic pathway and instead forms an ER-derived vacuole, while *C. burnetii* requires delivery to mature, acidified endosomes which it remodels into a large, replicative vacuole. Throughout infection, *L. pneumophila* effectors act at multiple points to inhibit recognition by xenophagy receptors and disrupt host autophagy, ensuring it avoids fusion with destructive lysosomes. In contrast, *C. burnetii* employs its effector cohort to control autophagy, hypothesized to facilitate the delivery of nutrients and membrane to support the growing vacuole and replicating bacteria. In this review we explore the effector proteins that these two organisms utilize to modulate the host autophagy pathway in order to survive and replicate. By better understanding how these pathogens manipulate this highly conserved pathway, we can not only develop better treatments for these important human diseases, but also better understand and control autophagy in the context of human health and disease.

## Introduction

Autophagy is an essential cellular pathway which, at its most basic, acts to degrade unwanted molecules and recover nutrients for the cell. Autophagy is classified into three broad categories: macroautophagy, microautophagy, and chaperone-mediated autophagy. Microautophagy involves the direct engulfment of cytoplasmic cargo by lysosomes and is largely non-specific (Li et al., 2012b), while chaperone-mediated autophagy entails the specific selection of cytosolic proteins by chaperones which directly target them to lysosomes for degradation (Kaushik and Cuervo, 2012). In contrast, macroautophagy (hereafter autophagy) involves the targeted degradation of molecules or organelles in the cell, which are first engulfed by a double-membraned autophagosome that later fuses with a proteolytic lysosome to degrade its contents. Autophagy is a fundamental and highly conserved process in all eukaryotes, with common autophagy proteins (ATG) found in plants, fungi, mammals, and amoeba (King, 2012). It is also involved in a myriad of essential cellular processes, including the elimination of damaged organelles and protein aggregates, nutrient recovery, and disease suppression (Codogno and Meijer, 2005; Nixon, 2013; Anding and Baehrecke, 2015; Ravanan et al., 2017; Yun and Lee, 2018; Wang et al., 2019).

Autophagy is also an important host defence against intracellular pathogens, where it acts to direct invading bacteria to autolysosomes for degradation (a process known as xenophagy) (Rikihisa, 1984; Gutierrez et al., 2004; Nakagawa et al., 2004). Perhaps expectedly, this has led to an evolutionary arms race between host and pathogens, which have developed a range of mechanisms to evade destruction. These host-pathogen interactions have been the subject of much research, providing valuable information not only about key virulence factors and important infections, but also about the underlying host molecular mechanisms that they subvert (Campoy and Colombo, 2009; Kimmey and Stallings, 2016; Casanova, 2017; McEwan, 2017; Sharma et al., 2018; Siqueira et al., 2018; Sudhakar et al., 2019; Wu and Li, 2019; Xiong et al., 2019; Hu et al., 2020).

**Figure 1:**
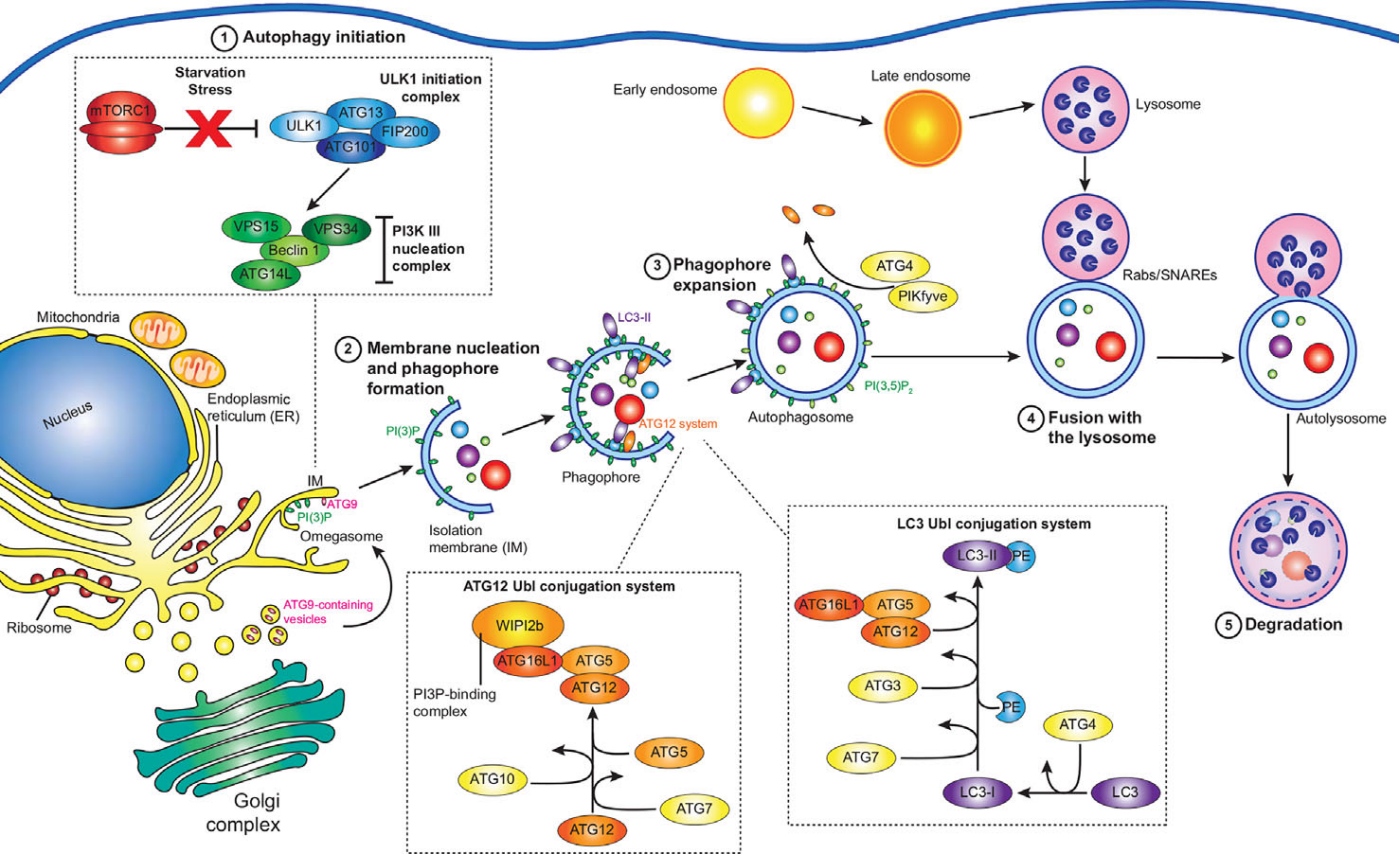
Summary of the autophagy pathway. Under normal conditions, active mTORC1 inhibits autophagy. However, under various stresses such as starvation, mTORC1 is inactivated, and autophagy initiation (1) can proceed. The ULK1 initiation complex consisting of ULK1, ATG13, FIP200, and ATG101 assembles, which then activates the PI3K complex I, containing VPS34, Beclin-1, VPS14, and ATG14L. Along with ATG9 containing vesicles, the PI3K complex I initiates the next step: membrane nucleation and phagophore formation (2). At isolation membranes (IM) on omegasomes at the ER, the PI3K complex I produces PI(3)P, while ATG9 vesicles promote membrane expansion into a phagophore. The ATG12 and LC3 ubiquitin-like (Ubl) conjugation systems act in concert to convert cytosolic LC3 to membrane bound LC3-II, mature LC3 conjugated to PE. WIPI2b recruits the ATG12 Ubl conjugation system to PI(3)P, leading to the accumulation of LC3-II at the growing phagophore, leading to the expansion and maturation of the phagophore (3). Autophagy receptors, such as SQSTM-1 bind targets for degradation and associate with LC3-II at the growing phagophore, which then closes around the cargo. ATG4 then removes ATG proteins from the outside of autophagosome, while PI(3)P kinases convert PI(3)P to PI(3,5)P_2_ to favour autophagosome maturation. The remaining LC3-II and PI(3,5)P_2_ promote the accumulation of Rab GTPases, membrane tethering complexes, and SNARES, which facilitate the fusion of autophagosomes to late endosomes/lysosomes (4). The autophagosome cargo is then exposed to the proteolytic and acidic contents of the lysosome, resulting in its degradation (5).

The autophagy pathway has been thoroughly reviewed previously (Glick et al., 2010; Bento et al., 2016; Yin et al., 2016; Dikic and Elazar, 2018; Yu et al., 2018; Levine and Kroemer, 2019; Wang et al., 2019), however a summary of key events is provided here (**Figure 1**). Autophagy is largely regulated by the mammalian target of rapamycin (TOR) complex 1 (mTORC1), which when active phosphorylates and inhibits autophagy initiation factors, but is inactivated by physiological stresses such as energy depletion, hypoxia, and starvation (Laplante and Sabatini, 2009; Rabanal-Ruiz et al., 2017). Upon the inactivation of mTORC1, Unc‐51‐like kinase 1 (ULK1) and ATG13 are dephosphorylated, forming a stable complex with focal adhesion kinase family‐interacting protein of 200 kDa (FIP200) and ATG101 to form the ULK1 complex (Kim et al., 2011). The active ULK1 complex then activates class III phosphatidylinositol 3-kinase (PI3K) complex I, consisting of the lipid kinase VPS34, Beclin-1, VPS15, and ATG14L, recruiting it and ATG9 to the isolation membrane (IM) - the precursor of the autophagosome membrane (Mack et al., 2012; Russell et al., 2013).

The PI3K complex I produces phosphatidylinositol 3-phosphate (PI(3)P) at the site of autophagosome formation by phosphorylating phosphatidylinositol (PI) (Ktistakis et al., 2012). While the exact source of the IM is unknown, it is likely that multiple sources contribute membrane (Chan and Tang, 2013). The association of the IM with markers of autophagosome initiation at cup-shaped sites at the ER, termed omegasomes, indicates that the ER plays an essential role (Ktistakis et al., 2008; Karanasios et al., 2013). The transient interaction of ATG9-containing vesicles at the IM promotes its expansion to a phagophore (Orsi et al., 2012), potentially *via* the delivery of lipids for membrane formation. Two ubiquitin-like (Ubl) systems then work in concert to attach lipidated LC3 (ATG8) proteins to the phagophore. The first system is the ATG12 Ubl conjugation system, consisting of ATG12, ATG7, ATG10, ATG5, and ATG16L1, results in the formation of an ATG12-ATG5-ATG16L1 complex which is recruited to PI(3)P positive membranes by WIPI2 (Polson et al., 2010; Dooley et al., 2014). The second system is the LC3 Ubl conjugation system, in which ATG4 cleaves the LC3 precursor to produce LC3-I, which is then activated by ATG3 and ATG7 before being conjugated to phosphatidylethanolamine (PE) by the ATG12-ATG5-ATG16L1 complex to produce the membrane associated LC3-PE (LC3-II) (Klionsky and Schulman, 2014).

Cargo destined for autophagic degradation are bound to LC3-II on the inner surface of the developing phagophore by a range of adaptor proteins which bind specific targets (Johansen and Lamark, 2020). In the case of xenophagy, invading pathogens are ubiquitinated by a range of E3 ligases, which acts as binding targets for adaptor proteins (Chen et al., 2019). Once cargo is bound, the crescent shaped phagophore closes to become an autophagosome. At this time ATG4 disassociates external ATG proteins and PI(3)P kinases convert PI(3)P to PI(3,5)P_2_, promoting maturation (Yu et al., 2012; Dall’Armi et al., 2013; Reggiori and Ungermann, 2017). Autophagosomes can fuse with endosomes or directly with lysosomes to create autolysosomes (Berg et al., 1998), where the cargo is degraded by the acidic and proteolytic environment provided by the lysosome. Fusion of the different vesicles is controlled by three protein groups: Rab GTPases, membrane-tethering complexes, and soluble N-ethylmaleimide-sensitive factor attachment protein receptors (SNAREs). Rab GTPases recruit tethering complexes to the target membranes and SNAREs are required for the fusion of the lipid membranes (Nakamura and Yoshimori, 2017; Lorincz and Juhasz, 2020).

*Coxiella burnetii* and *Legionella pneumophila* are related intracellular pathogens which take very different approaches to replicating within the host cell. Both are Gram-negative pathogens that possess a Dot/Icm Type IV secretion system (T4SS) which they use to translocate hundreds of effector proteins into the host and produce a replicative vacuole. *L. pneumophila* uses a subset of its effectors to inhibit autophagy and prevent delivery of the pathogen to lytic autolysosomes and in stark contrast, *C. burnetii* uses its effectors to co-opt host autophagy, using it to develop a vast replicative vacuole that can occupy the majority of the cellular space.

**Figure 2:**
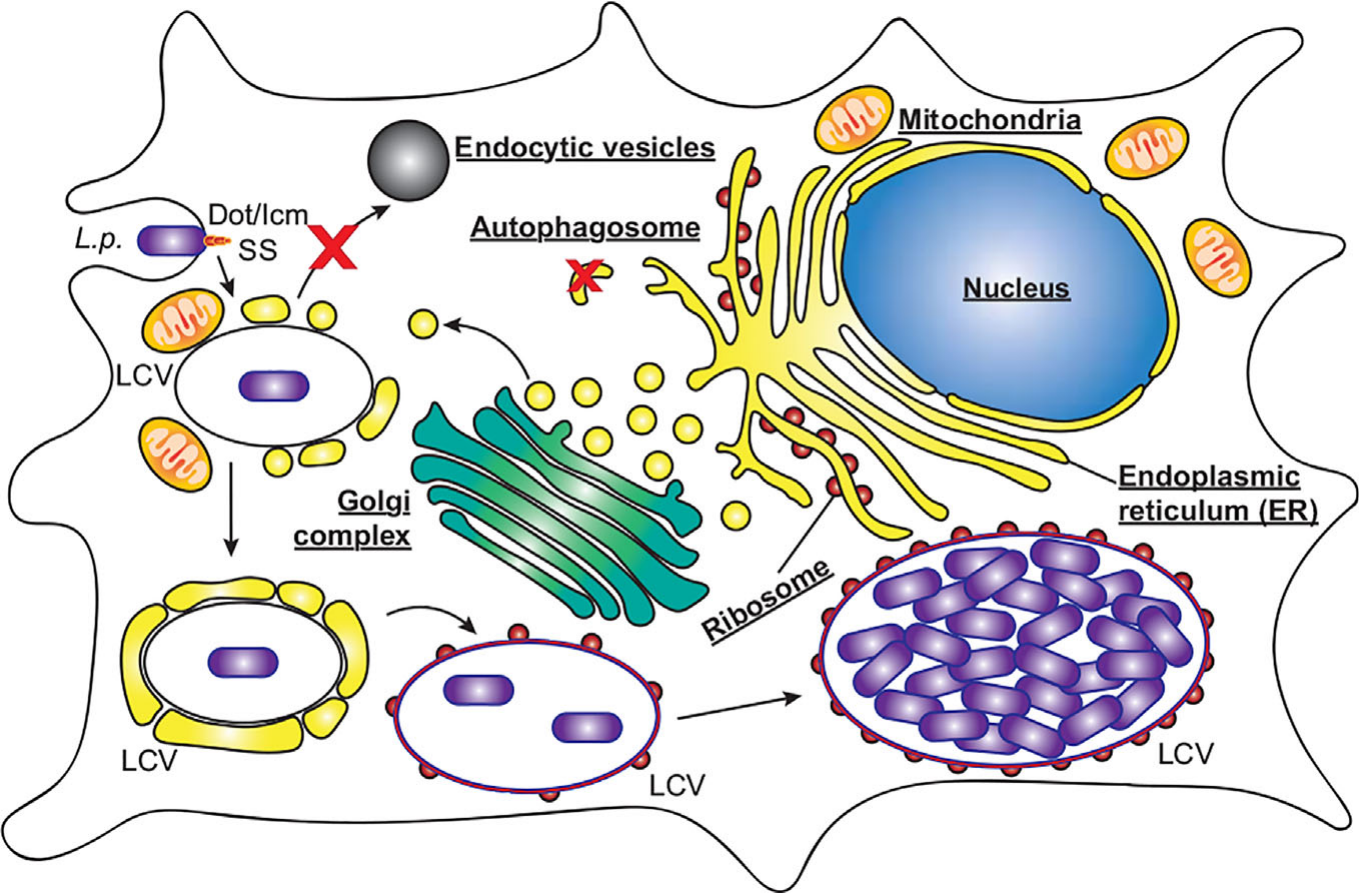
*L. pneumophila* replication within macrophages. *L. pneumophila* (*L.p.*) activates the Dot/Icm secretion system (SS) upon contact with macrophages, and following entry evades endocytic vesicles and autophagosomes to form a replicative niche termed the *Legionella*‑containing vacuole (LCV). Initially surrounded by vesicles derived from the endoplasmic reticulum and mitochondria, the LCV eventually resembles rough ER decorated with ribosomes, within which bacteria replicate to high numbers, eventually leading to cell lysis.

*L. pneumophila* is the causative agent of the severe pneumonia‑like disease, Legionnaires’ Disease. The environmental reservoir of *L. pneumophila* includes natural water sources, such as rivers and lakes, as well as man‑made sources, including, but not limited to, cooling towers and air‑conditioners, in which the bacteria resides within a wide range of protozoan hosts (Rowbotham, 1980). Opportunistic infection of humans occurs following the inhalation of contaminated aerosols. *L. pneumophila* enters the human host *via* phagocytic uptake by alveolar macrophages (Horwitz, 1983). Immediately, *L. pneumophila* begins to remodel the phagocytic vacuole, to avoid the host endocytic pathway and lysosomal degradation, by creating an ER-like replicative niche for replication, termed the *Legionella*‑containing vacuole (LCV, **Figure 2**) (Horwitz, 1983). A key virulence determinant in this process is its T4SS and the repertoire of over 300 bacterial effectors that are translocated into the host cell through this secretion system (Segal et al., 1998; Vogel et al., 1998; Gomez-Valero et al., 2011). Translocation of *L. pneumophila* effector proteins is initiated upon contact with the host cell plasma membrane (Chen et al., 2007; Charpentier et al., 2009), enabling them to immediately manipulate a range of host cell processes in a coordinated manner. Modulated systems include host vesicle trafficking pathways, ubiquitin machinery, autophagy and apoptosis, facilitating bacterial replication (Hubber and Roy, 2010; Isaac and Isberg, 2014; Prashar and Terebiznik, 2014; Ensminger, 2015; Omotade and Roy, 2019). The plasticity of the *Legionella* effector repertoire has been well documented, with comparative genomic studies revealing large variability in effector cohorts between *Legionella* species, with effectors largely unique to each species but presenting significant functional redundancy (Burstein et al., 2016; Gomez-Valero et al., 2019). This is likely a result of its association with a broad range of amoebal hosts, which would drive the evolution of an array of host specific genes that are redundant for infection in human macrophages (Park et al., 2020). Given the conservation of autophagy proteins within protozoan hosts (Calvo-Garrido et al., 2010), there is likely significant evolutionary pressure to evolve and maintain the subset of effectors that regulate host autophagy to facilitate bacterial success.

**Figure 3:**
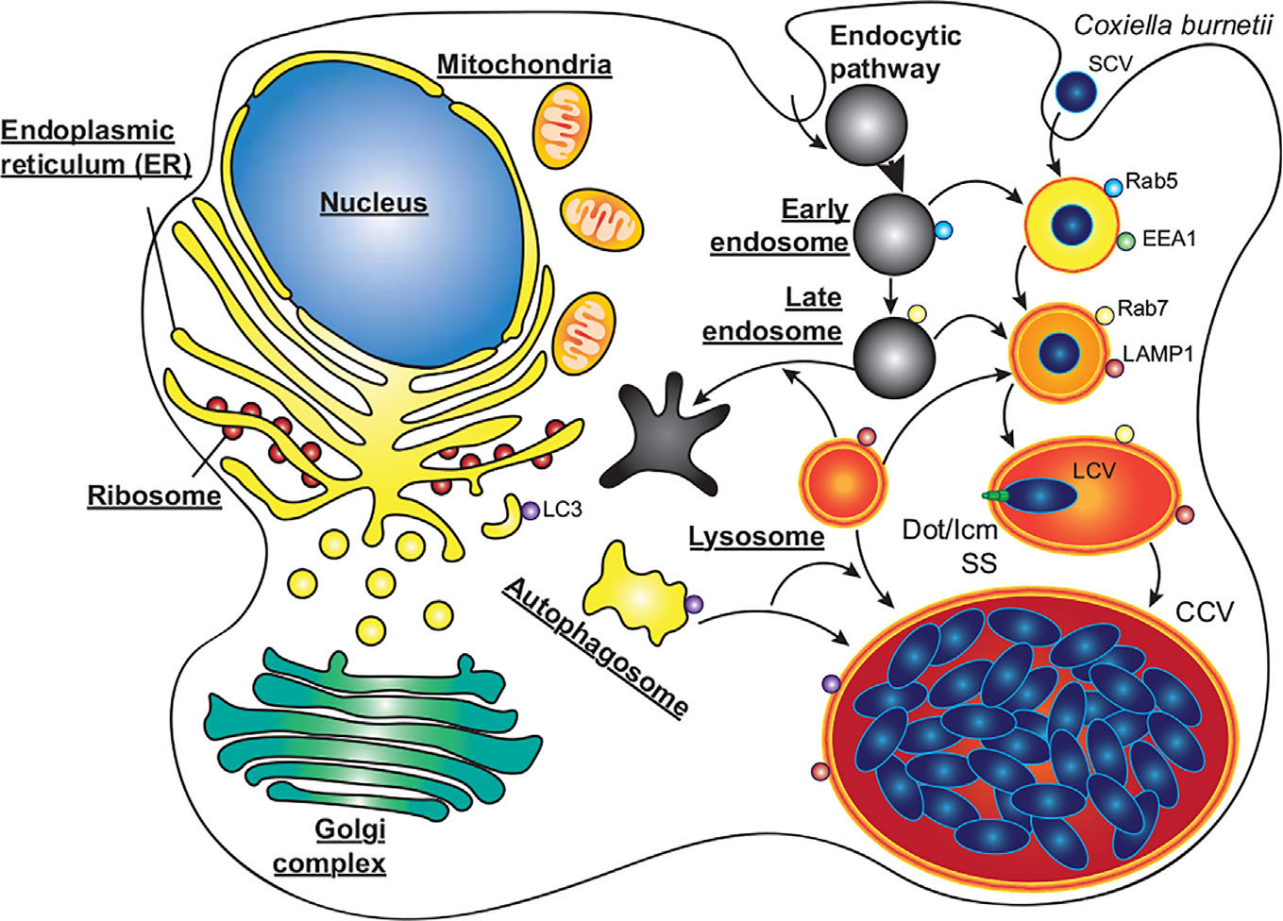
*C. burnetii* replication within macrophages. *C. burnetii* exists in two developmental stages; small cell variants (SCV) which are metabolically inactive but highly stable, and the metabolically active large cell variants (LCV). SCVs enter alveolar macrophages through phagocytosis and proceed through the endocytic pathway. Upon fusion of the pathogen containing phagosome with lysosomes, the SCVs convert to LCVs, and the Dot/Icm secretion system (SS) is activated. Through the activity of effector proteins, the mature phagolysosome is converted into the replication permissive *Coxiella* containing vacuole (CCV). The CCV rapidly expands through heterotypic fusion with autophagosomes, lysosomes, and endocytic vesicles, and homotypic fusion of multiple CCVs. As the CCV expands and fills with bacteria, the LCVs convert back to SCVs, where they will be stable upon release from the cell *via* cell lysis or exocytosis.

*C. burnetii* requires delivery to mature endosomes and fusion with lysosomes for a successful infection (Beron et al., 2002; Gutierrez et al., 2005). *C. burnetii* is the causative agent of Q fever, which although symptomatic in around 40% of cases, can develop into chronic Q fever in 1–5% of cases, with a mortality rate of up to 60% if left untreated (Kampschreur et al., 2015). *C. burnetii* is globally distributed, assisted by its high infectivity and broad host range including humans, fish, birds, arthropods, and livestock (Heinzen et al., 1999). As with *L. pneumophila*, *C. burnetii* infects alveolar macrophages where it enters the host cell by phagocytosis, and its pathogenicity also relies on a functional T4SS. However, *C. burnetii* does not avoid autophagy, instead passing through the host endocytic pathway where the pathogen-containing phagosome fuses with lysosomes. Only upon the subsequent acidification of the phagosome is the *C. burnetii* T4SS activated, translocating approximately 150 effector proteins to modify the host cell and develop the mature phagolysosome into the replication permissive *Coxiella*-containing vacuole (**Figure 3**, CCV) (Howe et al., 2003; Mahapatra et al., 2010; Newton et al., 2013; Burette and Bonazzi, 2020; Newton et al., 2020). The CCV is a spacious vacuole due to its highly fusogenic nature, with multiple CCVs in a cell fusing to form a single large vacuole that promiscuously fuses with host autophagosomes, lysosomes and endocytic vesicles. In this review we will discuss the role that autophagy plays during infections with *L. pneumophila* and *C. burnetii*, focusing on the effector proteins that they use to interfere with the host autophagy system (**Table 1**).

**Table 1:** *L. pneumophila* and *C. burnetii* effectors known to modulate autophagy.

Effector	Size: aa (kDa)	Known mechanism	Notes
***Legionella pneumophila* effectors**			
RavZ (Lpg1683)	502 (56.25)	Cysteine protease, cleaves LC3-II	Possesses PI(3)P binding domain and LIRs Found in 4/41 *Legionella* species^a^
*Lp*Spl (Lpg2176; Lpp2128; LegS2)	608 (67.38)	Degrades sphingolipids	Found in 10/41 *Legionella* species^a^
Lpg1137	322 (35.8)	Degrades Stx17	Found in 4/41 *Legionella* species^a^
Lpg2936	244 (27.29)	Methyltransferase, modifies host DNA	Found in 41/41 *Legionella* species^a^
LegA9 (Lpp2058)	580 (65.17)	Unknown	Found in 18/41 *Legionella* species^a^
SidE family (SidE, SdeA, SdeB, SdeC)	SidE – 1514 (171.69) SdeA – 1499 (169.08) SdeB – 1926 (216.99) SdeC – 1538 (172.93)	Creates non-canonical ubiquitin linkages	Found in 6/41 *Legionella* species^a^
Lgt1, Lgt2, Lgt3	Lgt1 – 525 (59.59) Lgt2 – 636 (71.65) Lgt3 – 873 (99.67)	Glucosylates and inhibits eEF1A	Lgt1 found in 3/41 *Legionella* species^a^ Lgt2/3 found in 1/41 *Legionella s*pecies^a^
SetA (Lpg2157)	1506 (169.78)	Glucosylates to activate TFEB	Found in 2/41 *Legionella* species^a^
***Coxiella burnetii* effectors**			
CvpA (CBU0665)	328 (38.01)	Interacts with AP2	Contains endocytic sorting motifs
CvpB (Cig2; CBU0021)	809 (93.1)	Prevents conversion of PI(3)P to PI(3,5)P_2_ by PIKfyve	
Cig57 (CBU1751)	420 (48.85)	Interacts with FCHO2	Contains endocytic sorting motifs
CvpF (CBU0626)	695 (79.58)	Interacts with Rab26	Contains endocytic sorting motifs

^a^(Burstein et al., 2016).

LIR, LC3 interacting region.

## Manipulation of Host Autophagy by *Legionella* Effectors

**Figure 4:**
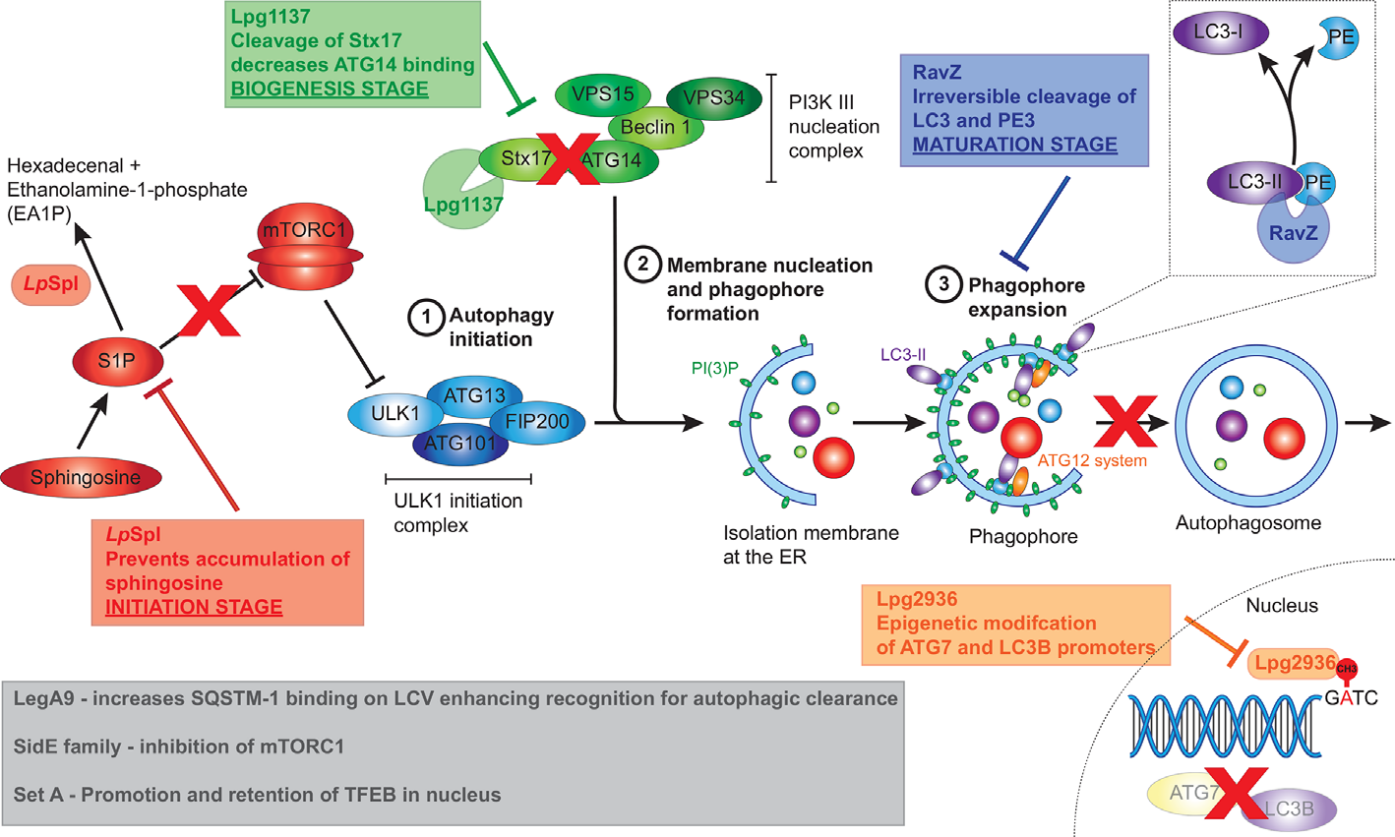
*L. pneumophila* effectors that manipulate host autophagy pathway. *L. pneumophila* effectors interfere with the host autophagy pathway at multiple stages including initiation, biogenesis and maturation stages. The conversion of spingosine‑1‑phosphate (S1P) to hexadecenal and ethanolamine‑1‑phosphate by *Lp*Spl prevents sphingosine accumulation resulting in mTORC1 activation, thereby inhibiting the initiation of autophagy. Lpg1137 acts to cleave Stx17, also interfering with the initiation of autophagy. RavZ interrupts the maturation process of autophagy through irreversible cleavage of PE from LC3-II. Finally, Lpg2936 results in epigenetic modification of key autophagy components, including ATG7 and LC3B, resulting in their decreased expression. Grey box indicates effectors that result in the initiation of autophagy, seemingly at odds with *L. pneumophila’s* intracellular niche. These including the SideE family of effectors which inhibits mTORC1, SetA which activates TFEB, and LegA9 which targets the LCV for autophagic recognition.

Intracellular replication of *L. pneumophila* is dependent on its ability to prevent the rapid fusion of the LCV with lysosomes, a process requiring a functional T4SS (Marra et al., 1992; Berger and Isberg, 1993; Roy et al., 1998; Wiater et al., 1998). *L. pneumophila* was found to associate with the autophagy proteins ATG7 and ATG8 rapidly after infection in a T4SS-dependent manner and to induce autophagosome formation in the host cell (Amer and Swanson, 2005; Amer et al., 2005). However, conflicting data has made it challenging to understand the importance of host autophagy during *L. pneumophila* infection. Early findings demonstrated that inhibition of autophagy, by treatment with the PI3K inhibitor 3-methyladenine (3-MA), increased the degradation of intracellular *L. pneumophila* (Amer and Swanson, 2005), while starvation induced autophagy was found to favour intracellular replication (Swanson and Isberg, 1995). A more recent study found upregulation of autophagy, using the glycolytic inhibitor 2‑deoxy‑glucose (2DG) or starvation inhibited *L. pneumophila* replication, while siRNA gene silencing of the autophagy protein ATG5 enhanced replication (Matsuda et al., 2009). It was later found that 2DG treatment did not affect bacterial replication in amoebae, and that *L. pneumophila* in macrophages were directly sensitive to 2DG through the hexose-phosphate transporter UhpC (Price et al., 2018). These studies suggest a complex interaction between *L. pneumophila* and the host autophagy pathway, encompassing both beneficial and detrimental aspects, that may be influenced by host specific factors and LCV maturation stage. However, given the observation that *L. pneumophila* actively inhibits host autophagy (Choy et al., 2012), it is likely that the degradative aspect of autophagy is detrimental. In support of this, many *L. pneumophila* effectors have now been identified which inhibit or modulate the host autophagy pathway (**Figure 4**).

### RavZ—Targeting LC3‑II to Inhibit Autophagy

The first *L. pneumophila* effector recognized as a manipulator of host autophagy was RavZ. RavZ was identified as the Dot/Icm effector responsible for reducing levels of the lipidated form of LC3 (LC3‑II) during infection, thereby inhibiting autophagy (Choy et al., 2012). Infection with RavZ-deficient *L. pneumophila* led to increased levels of LC3‑II and LC3‑postitive puncta within the host cells however intracellular bacterial replication was not impacted and vacuoles containing Δ*ravZ* mutant were not surrounded by LC3‑positive puncta. This provided foundational evidence that other effectors may be secreted by *L. pneumophila* to aid bacterial evasion of the host autophagy machinery (Choy et al., 2012). RavZ acts as a cysteine protease that specifically targets lipidated ATG8 family members, including LC3‑II. Cleavage of the amide bond between the aromatic tyrosine and the PE‑conjugated glycine residue by RavZ results in the irreversible deconjugation of LC3, since the reactive C‑terminal glycine normally required for conjugation of PE to LC3 has been removed, ultimately preventing autophagosome formation (Choy et al., 2012) (**Figure 4**). This reaction happens much more rapidly and efficiently compared to its eukaryotic host counterpart, ATG4. ATG4 is also a cysteine protease that deconjugates PE from LC3, although the cleavage occurs after the C‑terminal glycine, thereby allowing reconjugation of LC3.

Several research teams have solved the crystal structure of RavZ in order to fully elucidate how RavZ targets membrane‑associated ATG8 proteins and catalyses the irreversible deconjugation reaction (Horenkamp et al., 2015; Kwon et al., 2017; Yang et al., 2017). RavZ is composed of two domains, an N‑terminal catalytic domain that is responsible for cleavage of LC3‑II and a C‑terminal domain that binds to PI(3)P on membranes. Recognition of the LC3 substrate occurs through the LC3‑interacting region (LIR) motifs located at both the N‑ and C‑terminal regions of RavZ (Horenkamp et al., 2015; Kwon et al., 2017; Yang et al., 2017). Horenkamp et al. (2015) demonstrated that RavZ preferentially binds to high-curvature domains enriched with PI(3)P, commonly associated with phagophore membranes, and that the structural fold of the N-terminal catalytic domain is related to cysteine proteases of the Ubl‑specific protease (Ulp) family, with the catalytic triad of Cys258, His176 and Asp197 essential for the function of RavZ. Using numerous biochemical, structural and cell-based analyses, Kwon et al. (2017) observed that both the N- and C‑terminal contain LIR motifs that each bind LC3 suggesting that RavZ forms a complex with two molecules of LC3. These LIR motifs possess a higher binding affinity for LC3 than ATG4 and are critical for autophagy inhibition by RavZ. Significantly, Yang et al. (2017) generated semisynthetic LC3 proteins to solve the structure of the RavZ:LC3 interaction and proposed a “lift and cut” mode of action for deconjugation of LC3‑II by RavZ. Initially, RavZ targets PI(3)P associated with autophagosome membranes *via* its C‑terminal domain and a hydrophobic α3 helix and utilizes the N‑terminal LIR motif to bind LC3‑II. The α3 helix then aids extraction of the lipid PE moiety and docking of the fatty acid chain within the lipid‑binding site of RavZ. Both the LIR interaction and lipid binding are essential for the subsequent proteolytic cleavage of LC3‑II (Yang et al., 2017). Further clarification of the specific roles of the binding domains of RavZ was elucidated recently using a series of mutational analyses (Park et al., 2019). Park et al. (2019) observed that targeting autophagosome membranes occurs through two complementary independent pathways: either through PI(3)P-binding (found on early autophagic membranes) or through the LIR motifs binding LC3‑II (found on late autophagic membranes), thereby maximising the efficiency of RavZ to inhibit autophagosome formation. Notably, the absence of either domain did not influence the protease function of RavZ.

### LpSpl—Influencing Autophagy Through Sphingolipid Metabolism

Interestingly, *L. pneumophila* is also able to inhibit autophagy indirectly by interfering with host sphingolipid metabolism (Rolando et al., 2016). Sphingolipids, and their metabolites sphingosine, sphingosine‑1‑phosphate (S1P), ceramide and ceramide‑1‑phosphate, are important components of eukaryotic cell membranes and key signalling molecules involved in a number of host cell processes (Takabe et al., 2008). In particular the leve of S1P, which is tightly regulated by the enzyme sphingosine‑1‑phosphate lyase 1 (SGPL1), is critical for the balance between sphingolipid induced autophagy and cell death (Takabe et al., 2008).

The *L. pneumophila* effector *Lp*Spl exhibits similarity to eukaryotic SGPL1 in both sequence and activity and has been observed to localize to the mitochondria during infection (Degtyar et al., 2009). Elucidation of the crystal structure of *Lp*Spl confirmed a high level of structural conservation within both the core domain and active site when compared to human SGPL1, in particular, the position of 11 catalytically important residues (Rolando et al., 2016). The lyase activity of *Lp*Spl was confirmed during infection using an *Lp*Spl mutant in mouse embryonic fibroblasts (MEFs) missing endogenous Spl (MEF*spl^-/-^*), in which both wild‑type and complemented strains exhibited lyase activity, but not the Δ*spl L. pneumophila* strain. Additionally, mutations in key residues of the active site also abolished the enzymatic function of *Lp*Spl (Rolando et al., 2016). In contrast to previous observations that suggested *Lp*Spl localizes to the mitochondria (Degtyar et al., 2009), *Lp*Spl was also observed at the ER (Rolando et al., 2016). The observation of different subcellular localizations of ectopically expressed *Lp*Spl may reflect the different cell lines, expression levels and tags used in these studies. The localization of endogenous, *L. pneumophila* translocated *Lp*Spl remains to be determined.

Using mass spectrometry, Rolando et al. (2016) observed changes in host cell sphingolipid metabolism during infection with *L. pneumophila*. In particular, during wild‑type infection, levels of sphingomyelin, ceramide and glycosphingolipids were decreased compared to uninfected cells, although this was not dependent on *Lp*SpI. Cellular levels of sphingosine, however, were significantly increased during infection with the Δ*spl* strain compared to wild‑type and complemented strains. Given that translocation of *Lp*Spl by *L. pneumophila* prevents accumulation of sphingosine during infection, and sphingosine stimulates autophagy (Dall’Armi et al., 2013), the impact of *Lp*Spl on the host autophagy machinery was investigated further. Depletion of LC3‑II during the ectopic expression of *Lp*Spl under starvation conditions, and no difference in the ratio between autophagosomes and autolysosomes, suggested that *Lp*Spl prevents autophagosome biogenesis, and not autophagosome maturation like RavZ. This action was dependent on the enzymatic function of *Lp*Spl as catalytically inactive mutants did not decrease host autophagy activity (Rolando et al., 2016). Finally, an increase in LC3 puncta was observed during infection of host cells with *L. pneumophila* Δ*spl* compared to wild‑type. This increase in LC3 puncta was not as pronounced as the Δ*ravZ* mutant and the *Δspl/ravZ* double mutant strains, with RavZ contributing more to autophagy inhibition than *Lp*Spl (Rolando et al., 2016). Deletion of *Lp*Spl did not impact intracellular bacterial replication in amoeba or macrophages, similar to RavZ (Choy et al., 2012), but *Lp*Spl is required for efficient replication in a mouse model of infection (Rolando et al., 2016). Ultimately, secretion of *Lp*Spl interferes with S1P, limiting autophagosome biogenesis in the host cell and aiding bacterial survival (**Figure 4**).

### Lpg1137—Eliminating the Role of Syntaxin 17

The incidental observation that Syntaxin 17 (Stx17) is degraded during *L. pneumophila* infection in a T4SS-dependent manner led to the discovery of Lpg1137 as the responsible effector (Arasaki et al., 2017). Stx17 is a SNARE protein initially implicated in vesicle trafficking (Steegmaier et al., 2000) but which also has a role in autophagy (Hamasaki et al., 2013; Diao et al., 2015; Kumar et al., 2019; Xian et al., 2019). Stx17, along with ATG14L, SNAP29 and VAMP8, facilitates the fusion of autophagosomes with lysosomes to produce mature autolysosomes (Itakura et al., 2012; Diao et al., 2015; Shen et al., 2020). Additionally, Stx17 has been shown to promote the assembly of the ULK1 initiation complex (Kumar et al., 2019), and has also been found to recruit ATG14L to the mitochondria-associated ER membrane (MAM), which has been implicated in mitophagy (the autophagic degradation of mitochondria) (Xian et al., 2019) and phagophore initiation (Hamasaki et al., 2013). However, the exact role of Stx17 in autophagy initiation is still not entirely known.

Lpg1137 was demonstrated to be a serine protease that localizes to MAM, microsomes, and mitochondria, where it specifically interacts with the cytoplasmic C‑terminal of Stx17 (Arasaki et al., 2017). The degradation of Stx17 during *L. pneumophila* infection results in the blockage of autophagosome biogenesis in starvation‑induced autophagy (**Figure 4**). The formation of both ATG14‑positive and LC3‑positive puncta is inhibited in the presence of ectopically expressed Lpg1137 suggesting a failure of PI(3)P formation on omegasomes, similar to silencing Stx17 using siRNA (Arasaki et al., 2017). Despite this, deletion of *lpg1137* did not negatively impact intracellular bacterial replication (Arasaki et al., 2017).

Curiously, bioinformatic analysis and 3D‑structure modelling of Lpg1137 suggest this *L. pneumophila* effector is in fact a homologue of mitochondrial SLC25 carrier proteins (Gradowski and Pawlowski, 2017). The authors propose that the cleavage of Stx17 observed by Arasaki *et al*. occurs through either indirect or direct activation of an alternative serine protease in the mitochondrial inner membrane by Lpg1137, or that interaction with Lpg1137 may make Stx17 more prone to cleavage from endogenous proteases or other effector proteases (Gradowski and Pawlowski, 2017). Ultimately, solving the crystal structure of Lpg1137 will be required to fully elucidate its molecular function within host cells during infection.

### Lpg2936—Epigenetic Modulator of Autophagy Components

The *L. pneumophila* effector Lpg2936 has recently been implicated as a regulator of autophagosome formation (Abd El Maksoud et al., 2019). This finding arose from the observation that during *L. pneumophila* infection, the expression of autophagy-related genes ATG7 and LC3B was reduced, alongside a decrease in the expression of unlipidated LC3‑I, lipidated LC3‑II, and the ATG5-ATG12-ATG16L1 protein complex. Using RNAi against Lpg2936, investigators were able to restore expression of these autophagy‑related genes during *L. pneumophila* infection and consequently inhibited bacterial replication (Abd El Maksoud et al., 2019). Bioinformatic and structural analysis previously established that Lpg2936 is a ribosomal RNA protein similar to RsmE‑like methyltransferases (Pinotsis and Waksman, 2017). Subsequent examinations demonstrated that Lpg2936 is translocated into the host nucleus where it recognizes the GATC motif in the promoter regions of ATG7 and LC3B and induces irreversible methyladenine changes in this motif from GATC to G(6 mA)TC (Abd El Maksoud et al., 2019). Similar to RNAi against Lpg2936, methylation inhibitors reduced *L. pneumophila* replication and restored expression of autophagy‑related genes (Abd El Maksoud et al., 2019). Collectively, this data suggests that Lpg2936 is a transcription factor that translocates into the host cells to regulate autophagosome formation through epigenetic modification of ATG7 and LC3 promoter regions thereby enhancing bacterial replication (**Figure 4**). However, it is worth noting that Lpg2936 may have an autophagy independent role within the bacteria, and therefore silencing Lpg2936 using RNAi or the use of methylation inhibitors has a confounding impact on bacterial viability and replication. Indeed, Pinotsis and Waksman (2017) suggest that the RsmE fold of Lpg2936 is highly specific for bacterial 16S RNAs and would likely target the *Legionella* 16S RNA subunit, rather than eukaryotic ribosomes, enhancing the ability of the bacterium to produce large amounts of effectors during infection. As such, the modification of both ATG7 and LC3 could be inadvertent and unrelated to an effector role for Lpg2936.

### LegA9—the Odd One Out

Paradoxically, the *L. pneumophila* genome also encodes for a T4SS effector, ankyrin‑containing protein LegA9, that enhances recognition of the LCV for autophagy uptake and clearance (Khweek et al., 2013). The autophagic adapter SQSTM-1 (p62), which binds to both LC3 on autophagosomes and ubiquitinated cargo, is important for targeting intracellular material to the lysosome for clearance (Komatsu et al., 2007; Kirkin et al., 2009) (**Figure 4**). Deletion of *legA9* led not only to a decrease in lysosomal fusion of LCVs but also a reduction in the accumulation of ubiquitin labelling and SQSTM-1 at LCVs compared to wild-type, ultimately promoting bacterial replication within human and mouse-derived macrophages—the latter of which is normally restrictive to *L. pneumophila* replication (Khweek et al., 2013). Although the presence of LegA9 labels the *L. pneumophila* vacuole for autophagy uptake through SQSTM-1 binding, LegA9 does not have a direct role in autophagy activation as no difference in LC3‑II levels was observed between wild‑type and mutant strains following the stimulation of autophagy by rapamycin treatment (Khweek et al., 2013). Further, the increase in replication of the LegA9 mutant was eliminated by treatment with rapamycin. It is possible that, as with the *L. pneumophila* effector LamA, LegA9 may be adapted to favour infection in alternate hosts such as amoebae, and its induction of autophagy in mammalian cells may be an unintended consequence (Price et al., 2020).

### Opposing Roles for the SidE Family

The observation of enhanced SQSTM-1 binding on LCVs has recently been expanded on in a study by Omotade and Roy (2020). Investigations into whether ubiquitin‑marked LCVs recruit the necessary adapter receptors for autophagy revealed that despite enrichment of ubiquitin on the LCV, autophagy adapters such as SQSTM-1, NBR1, optineurin and NDP52 are largely absent from the LCV (Omotade and Roy, 2020). The authors proposed that the unique non‑canonical ubiquitin linkage created by the SidE family of effectors on proteins on the LCV (Bhogaraju et al., 2016; Qiu et al., 2016), prevents recognition by autophagy adapters. Indeed, a mutant in which all four family members (SidE, SdeA, SdeB, and SdeC) were absent resulted in the increased recruitment of SQSTM-1 to the LCVs. However, no significant increase in LC3B localization to the LCV was detected, even in the absence of RavZ confirming that other mechanisms exist in *L. pneumophila to* block xenophagy (Omotade and Roy, 2020). Additionally, co‑infection studies with *Listeria monocytogenes* revealed that the ability of the SidE family to exclude SQSTM-1 occurs specifically at the LCV and does not impact the ability of the cell to target *L. monocytogenes* for autophagic removal through SQSTM-1 binding.

Previously, in addition to the ubiquitylation activities of the SidE family, an effector screen identified the ability of SidE, SdeA, SdeB, and SdeC to promote nuclear translocation of TFEB (transcription factor EB) through inhibition of mTORC1 (De Leon et al., 2017). Active mTORC1 phosphorylates TFEB resulting in retention of TFEB within the cytosol. However, during nutrient limitation, mTORC1 is inactive and the subsequent dephosphorylated TFEB is translocated into the nucleus to induce the transcription of autophagic and lysosomal genes, thereby increasing nutrient availability (Settembre et al., 2012; Roczniak-Ferguson et al., 2012). The activation of TFEB by the SidE family of effectors seems incongruous to the action of other *L. pneumophila* effectors, especially in light of the same effector screen also identifying a role for the Lgt family of effectors (Lgt1, Lgt2, and Lgt3) in preventing TFEB translocation into the nucleus (De Leon et al., 2017). The Lgt family were previously identified as glucosyltransferases that inhibit translation by targeting host elongation factor 1A (eEF1A) (Belyi et al., 2006; Belyi et al., 2008). The authors argue that the two opposing families work together to provide enough nutrients for bacterial replication without promoting autophagy. The SidE family directly ubiquitinylate the Rag small‑GTPases that are necessary for mTORC1 to respond to elevated levels of amino acids, effectively blinding mTORC1 to the amino acids liberated by translation inhibitors such as the Lgt effector family (De Leon et al., 2017).

Interestingly, when co‑expression of both Lgt and SidE effectors was attempted, expression of SidE was blocked, suggesting temporal regulation of effector translocation is necessary for these effectors to function synergistically during infection (De Leon et al., 2017). Ultimately, inhibition of mTORC1 results in the induction of autophagy; however, secretion of effectors such as RavZ and *Lp*Spl ensure that autophagy is inhibited allowing *L. pneumophila* to acquire nutrients for replication without any detriment to survival. Extensive study of *L. pneumophila* manipulation of Rab1 has demonstrated that a small subset of effectors can control every aspect of Rab1 activity and localization, with effector pairs displaying opposing functions (Murata et al., 2006; Ingmundson et al., 2007; Machner and Isberg, 2007; Müller et al., 2010; Mukherjee et al., 2011; Neunuebel et al., 2011; Tan et al., 2011; Tan and Luo, 2011). Therefore it is possible other effectors can advantageously temporally regulate other key host processes including autophagy.

### SetA—Providing Nutrient Control

Recently a similar screen identified a cohort of effectors that promoted translocation of TFEB into the nucleus, including members of the SidE family, SdeA (Lpg2157) and SdeC (Lpg2153) confirming results observed by De Leon et al. (2017), as well as identifying novel effectors: MavH (Lpg2425), VipD (Lpg2831), Lpg2552, Lpg2828, Lpg2888, and SetA (Lpg1978) (Beck et al., 2020). SetA is a mono‑*O*‑glucosyltransferase containing a DxD catalytic motif that preferentially uses UDP‑glucose as a sugar donor targeting a range of host proteins including the small GTPase Rab1a, the chaperonin CCT5 and actin (Jank et al., 2012; Wang et al., 2018; Levanova et al., 2019; Gao et al., 2019). Beck et al. (2020) show that translocation of TFEB into the nucleus is dependent on the glucosyltransferase activity of SetA and mass spectrometry revealed the sites on TFEB that are modified by SetA. In particular, modification of S138 prevented nuclear export (and hence retention within the nucleus) and modification of a cluster of serine and threonine residues near the binding site of 14-3-3 prevents interaction between TFEB and 14-3-3, inhibiting the cytoplasmic retention of TFEB. Despite this, it is currently unknown what role SetA may play during infection, as these experiments were performed exogenously and not in the context of infection.

With the *Legionella* genus predicted to contain >18,000 effectors (Gomez-Valero et al., 2019), it is unsurprising that a significant number have been implicated in influencing autophagy. The high amount of functional redundancy observed in this extensive effector cohort poses a continuing challenge to understanding their collective temporal actions, as well as making it increasingly difficult to identify new effectors modulating this pathway.

## Manipulation of Host Autophagy by *Coxiella* Effectors

Following entry into a host cell, *C. burnetii* traffics through the endocytic pathway to an acidified mature endosome, activating its T4SS to facilitate development of the highly fusogenic CCV (**Figure 3**) (Beron et al., 2002; Romano et al., 2007; Newton et al., 2013; Newton et al., 2020). The CCV also accumulates markers of autophagosomes and lysosomes (Heinzen et al., 1996; Beron et al., 2002; Romano et al., 2007), as well as factors involved in their transport and fusion (Campoy et al., 2011; Campoy et al., 2013; McDonough et al., 2013).

Activation of host autophagy by starvation or rapamycin has been shown to increase the replication and viability of *C. burnetii* following infection (Gutierrez et al., 2005), as well as increasing the size of CCVs (Latomanski and Newton, 2018; Larson et al., 2019). Conversely, inhibition of autophagy by 3-MA impairs the development of the CCV (Beron et al., 2002; Latomanski and Newton, 2018), as does preventing vacuole acidification with bafilomycin A_1_ or chloroquine (Heinzen et al., 1996; Newton et al., 2013). Additionally, the essential autophagy genes ATG5, ATG7, ATG12 and STX17 are required for homotypic fusion into one large CCV (McDonough et al., 2013; Newton et al., 2014; Martinez et al., 2016).

**Figure 5:**
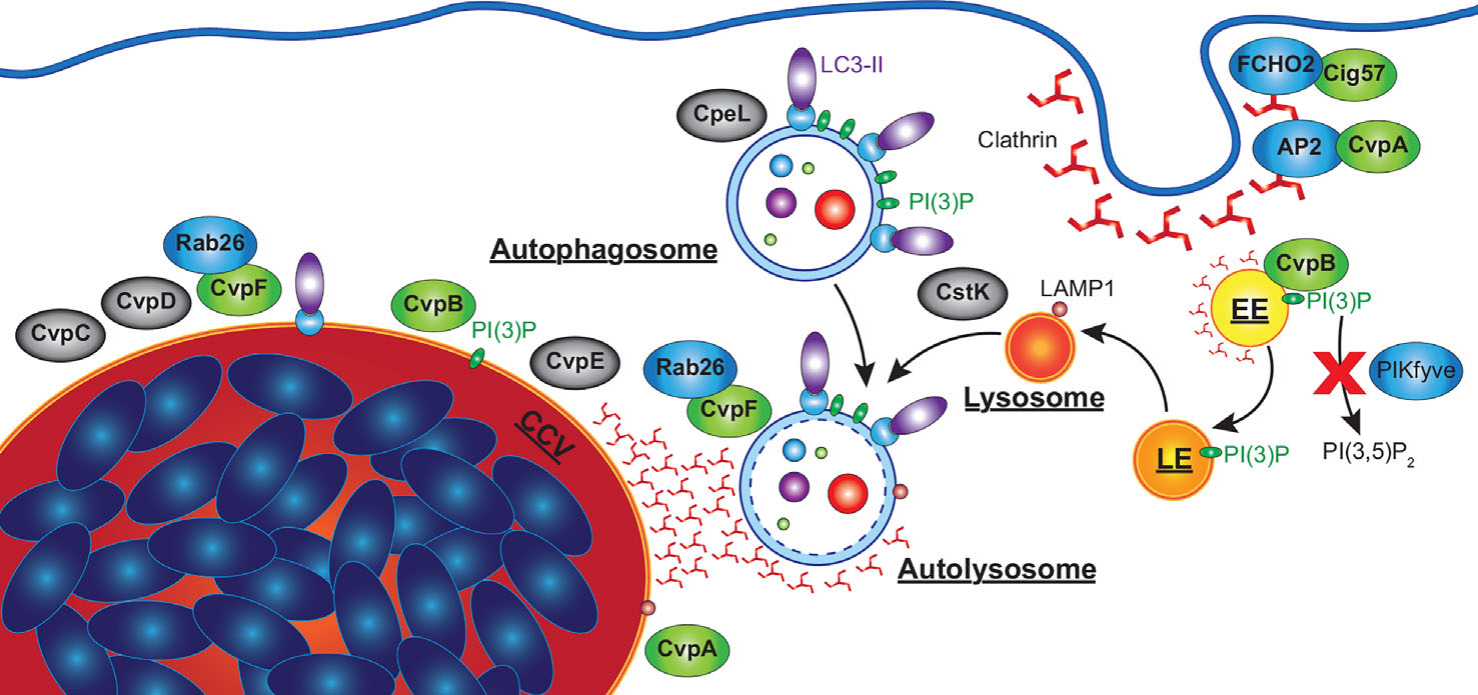
*C. burnetii* effectors that manipulate host autophagy pathway. Effectors of *C. burnetii* co-opt the autophagy pathway to facilitate the development of an extensive CCV. CvpA interacts with AP2, an adaptor involved in clathrin-mediated endocytosis (CME), and is required for CHC accumulation at the CCV. Cig57 associates with FCHO2, another protein involved in CME, and also promotes CHC accumulation at the CCV, as well as being required for *C. burnetii*-induced LC3 lipidation and the delivery of LC3-II positive vesicles to the CCV. CvpB associates with early endosomes and is essential for homotypic fusion of the CCV as well as the accumulation of LC3 at the CCV. CvpB increases cellular PI(3)P by preventing PIKfyve phosphorylating PI(3)P to produce PI(3,5)P_2_, facilitating the fusion of autophagosomes with the CCV. CvpF is also required for *C. burnetii*-induced LC3 lipidation, CCV formation, and LC3 delivery to the CCV *via* interactions with Rab26. CvpC, CvpD and CvpE associate with the CCV, and are required for its expansion, CpeL colocalizes with autophagosomes, and CstK was found to localize to vesicles and the CCV. These effectors (grey) have no confirmed activity, but their sub-cellular localizations suggests they may also manipulate autophagy. EE, early endosome; LE, late endosome.

While the importance of autophagy to *C. burnetii* is well established, the consequences of host-pathogen interactions on this pathway are less clear. Infection with *C. burnetii* induces an increase in total and lipidated LC3 indicative of an induction of autophagy, although infection also results in an increase in SQSTM-1 (Winchell et al., 2014; Latomanski and Newton, 2018; Larson et al., 2019). The latter finding is curious given that degradation of SQSTM-1 is often monitored as a read out for autophagic flux. However, starvation during infection can still induce autophagic degradation as seen by a decrease in SQSTM-1 (Latomanski and Newton, 2018; Larson et al., 2019). This suggests a more nuanced interference by *C. burnetii* than overt hyperactivation of autophagy or inhibition of autophagic degradation. Interestingly, while it is often hypothesised that *C. burnetii* induces autophagy to delivery nutrients to the expanding CCV, the difficulties in separating out this activity from other actions of autophagy has left this hypothesis without incontrovertible proof. Recent studies have, however, identified several *C. burnetii* effectors proteins involved in the modulation of autophagy (**Figure 5**), although this pathway is likely targeted by more effectors that remain to be characterized.

### CvpB—Stabilizing PI(3)P to Facilitate CCV Fusogenicity

One of the most thoroughly characterised *C. burnetii* effector proteins is *Coxiella* vacuolar protein B, named for its association with the CCV membrane (CvpB, Cig2, CBU0021). CvpB was initially identified in a transposon screen as essential for the normal biogenesis of the CCV (Newton et al., 2014). In contrast to the single large CCV of WT *C. burnetii*, CvpB-deficient bacteria instead formed multiple small vacuoles, suggesting a role in the homotypic fusion of CCVs. Interestingly, replication of CvpB transposon mutants was not impaired during infection of HeLa cells, although CvpB mutants were better tolerated by the *Galleria mellonella* infection model (Kohler et al., 2016). Despite replicating to similar numbers as WT, CvpB-deficient *C. burnetii* induced slower *Galleria* death. This is a significant observation that may implicate autophagy in disease pathology associated with *C. burnetii* infection.

A multivacuolar phenotype, similar to that of the *cvpB* mutant, was observed with gene silencing of Stx17 (McDonough et al., 2013). Silencing of key autophagy factors ATG5 and ATG12 also produced a multivacuolar phenotype, indicating that a functional host autophagy system is required for homotypic fusion of CCVs (Newton et al., 2014; Kohler et al., 2016). Likewise, *C. burnetii* engineered to translocate the *L. pneumophila* effector RavZ, which inhibits autophagy by cleaving lipidated LC3, also produced a multivacuolar phenotype. LC3 has been observed to be associated with CCVs, indicating the interaction of autophagosomes with the CCV and defining an autolysosomal state for the mature CCV (Beron et al., 2002; Kohler et al., 2016). However, in CvpB transposon mutants LC3 was absent from CCVs, suggesting that CvpB is required for autophagosome fusion with CCVs. CvpB did not interfere with autophagic flux, the delivery of endocytic cargo to the CCV, nor reduce the hydrolytic activity of CCVs (Newton et al., 2014). This indicates that CvpB acts to facilitate the fusion of CCVs and autophagosomes, but does not modulate autophagy itself.

The subcellular localization of ectopically expressed CvpB was examined in high detail using immuno-electron microscopy confirming its localization to the membrane of early endosomes (**Figure 5**). Based on this observation, *in vitro* assays were performed which identified PI(3)P as a binding target for CvpB (Martinez et al., 2016). CvpB lacks any predicted lipid-binding domains but mutational analysis identified the first 500 amino acids as essential for its membrane localization, and that PI(3)P was enriched at the CCV membrane in a CvpB-dependent manner. It was also found that, even in the presence of inhibitors of PI3K, PI(3)P was still detected on vacuolar structures (Martinez et al., 2016). While not a PI3K itself, CvpB instead was shown to inhibit the activity of the PI3 phosphate 5-kinase PIKfyve, which phosphorylates PI(3)P to produce PI(3,5)P_2_. Consistent with this, siRNA silencing of PIKfyve was able to correct the multivacuolar phenotype observed in CvpB transposon mutants. Expression of CvpB was found to disassociate PIKfyve from endosomes where it would normally bind and act upon PI(3)P, while CvpB with mutations in the membrane binding domain (MBD) were unable to displace PIKfyve. However, the MBD alone (aa 1-500) was also unable to relocate PIKfyve, suggesting that CvpB does not simply outcompete it for binding to PI(3)P (Martinez et al., 2016). Subsequent studies also found that CvpB is essential for the accumulation of clathrin heavy chain (CHC) at the CCV (Latomanski and Newton, 2018). Interestingly, in the absence of CvpB, clathrin was found associated with LC3B positive autophagosomes near the CCV, which did not fuse with the CCV (Latomanski and Newton, 2018). It is not currently known how CvpB prevents PIKfyve recruitment to PI(3)P positive membranes leading to the accumulation of PI(3)P, nor how this leads to the fusogenic CCV characteristic of *C. burnetii*. However, increased PI(3)P may stabilize LC3 or other pro-fusion molecules (i.e. SNARES) on the CCV membrane, which are normally absent from mature autolysosomes.

### Cig57—Enhancing LC3B Lipidation and Clathrin Localization at the CCV

Another effector identified through a transposon mutant screen for CCV defects is Cig57 (CBU1751). Disrupting Cig57 was found to impair the intracellular replication of *C. burnetii* and produce a small CCV phenotype (Newton et al., 2014). Cig57 contains multiple endocytic sorting motifs (two dileucine (DiLeu) and one tyrosine (Tyr)), suggesting it may also interact with clathrin-mediated endocytosis. Indeed, a yeast two-hybrid screen identified FCHO2, a protein that acts to curve the plasma membrane to initiate the formation of clathrin coated vesicles (Henne et al., 2010), as a binding partner of Cig57 (**Figure 5**) (Latomanski et al., 2016).

The identification of FCHO2 suggested an involvement of clathrin, and indeed CHC localization at the CCV was dependent on Cig57 containing functional endosomal sorting motifs (Latomanski et al., 2016). Interestingly, the adapter protein FCHO2 did not alter its sub-cellular localization in the presence or absence of Cig57. FCHO2 knock-out cells presented reduced, but not absent, CHC accumulation at the CCV, suggesting that while FCHO2 enhances the CCV accumulation of CHC, it is not essential. Immunofluorescence imaging found that CHC concentrated in areas where LC3B positive autophagosomes met the CCV, while siRNA silencing of CHC prevented the accumulation of LC3B at CCVs (Latomanski and Newton, 2018). Interestingly, in Cig57 mutants there were no LC3B positive vesicles associated with CHC at the CCV (Latomanski and Newton, 2018). This suggests that clathrin is required for the fusion of autophagosomes to the CCV, while Cig57 is required for the delivery of LC3B positive vesicles to the CCV.

Cig57 was also found to be essential for the *C. burnetii*-dependent LC3B lipidation observed during infection, although Cig57 alone was unable to induce LC3B lipidation (Latomanski and Newton, 2018). Furthermore, *C. burnetii* lacking Cig57 did not accumulate SQSTM-1 during infection as seen in WT strains. While starvation reduced the levels of SQSTM-1 in WT infections to that of non-infected cells, no change was observed in Cig57 transposon mutants. The size of Cig57 transposon mutant CCVs were also not altered by starvation-induced autophagy, while WT CCVs more than doubled (Latomanski and Newton, 2018). CCVs in HeLa cells with siRNA silenced CHC or Stx17 were also unresponsive to induced autophagy. This not only highlights the importance of autophagy to *C. burnetii* infection, but also points to a central role of CHC in mediating autophagy and Cig57 in exploiting this role to support CCV expansion. However, it is currently unclear how Cig57 facilitates LC3 lipidation, SQSTM-1 accumulation, or the fusion of CHC and autophagosomes to the CCV.

### CvpF—Inducing LC3 Lipidation *via* Rab26

More recently, an additional *C. burnetii* vacuolar protein, CvpF (CBU0626) was identified as important for intracellular replication of *C. burnetii* and CCV formation while being dispensable for replication in axenic medium (Siadous et al., 2020). During infection of U2OS osteosarcoma cells, both WT and CvpF transposon mutant *C. burnetii* strains developed acidified CCVs decorated with LAMP1, however CCVs produced by CvpF mutants were deficient in LC3B. Further, CvpF transposon mutants did not induce an increase in lipidated LC3B as seen in WT infections (Siadous et al., 2020). However, unlike Cig57, SQSTM-1 was still increased during infection with CvpF transposon mutants and reduced by starvation-induced autophagy. Ectopically expressed CvpF was found to partially co-localize with endosomal sorting complex required for transport (ESCRT), LAMP1, LC3B, and PI(3)P at compartments clustered around the nucleus. Ectopically expressed CvpF was also able to increase LC3B-II and SQSTM-1 levels in bafilomycin A_1_ treated cells, indicating an induction of autophagy. Sequence analysis identified three endocytic sorting motifs, of which a single Tyr motif was responsible for CvpF membrane localization as well as inducing LAMP1 and LC3B repositioning to vacuoles. Finally, transfection experiments visualized CvpF localized only to acidified autolysosomes, suggesting a role in autolysosome development (**Figure 5**).

A yeast two-hybrid screen identified the GTPase Rab26 as a binding partner of CvpF, with fluorescence microscopy confirming their colocalization and also observing that CvpF increases the membrane targeting of Rab26 (Siadous et al., 2020). CvpF was found to promote the accumulation of Rab26 at the CCV, although low level accumulation was still observed in CvpF mutants. Rab26 is involved in lysosomal positioning, autophagosome maturation, and the degradation of synaptic vesicles, and has been shown to interact with ATG16L1 (Li et al., 2012a; Jin and Mills, 2014; Binotti et al., 2015). Cells expressing dominant-negative forms of Rab26 possessed reduced LC3 at the CCV, while Rab26 knock-out cell lines supported smaller CCVs and impaired *C. burnetii* intracellular replication (Siadous et al., 2020). Additionally, exogenous expression of CvpF induced the formation of LC3B positive endosomes in transfected cells, which was inhibited by the co-expression of dominant negative Rab26. This data suggests that CvpF interacts with Rab26 to stimulate the accumulation of LC3B at the CCV. However, whether CvpF alters the sub-cellular localization of ATG16L1 or other factors involved in LC3B lipidation or autophagosome formation is currently unknown.

### Other Putative Autophagy Modulating Effectors

One of the first CCV-associated effector proteins identified by Larson and colleagues, CvpA, was identified from the *C. burnetii* genome due to the presence of multiple eukaryotic endocytic sorting motifs (Larson et al., 2013). As with other important effector proteins, replication of a CvpA mutant was not compromised in axenic medium, but was significantly reduced during intracellular replication in THP-1 macrophage-like cells (Larson et al., 2013). Additionally, CCVs produced by CvpA mutants were significantly smaller than WT C*. burnetii*. Ectopically expressed CvpA was observed to colocalize at the plasma membrane with CHC and early endosome antigen 1 (EEA1), at pericentrosomal vesicles labelled with LAMP1 (sorting endosomes and recycling endosomes; SE, RE), and in infected cells at the CCV membrane (**Figure 5**). At the plasma membrane, CvpA was found to associate with the transferrin (Tf) receptor (TfR) and inhibited the clathrin mediated uptake of Tf. Following up on this observation, it was demonstrated that CHC accumulated at the CCV in a CvpA-dependent manner.

CvpA contains a leucine-rich repeat associated with protein-protein interactions, and three DiLeu and two Tyr endocytic sorting motifs. Pull-down assays found that the clathrin adapter protein AP2, but not AP1 or AP3, bound to all DiLeu motifs and a peptide containing both Tyr motifs, while CHC was found to associate only to the Tyr-containing peptide, likely mediated by AP2. AP2 is an adaptor found exclusively at the plasma membrane and is involved in clathrin-mediated endocytosis. Cargo recognized by AP2 include the autophagosome initiation factors ATG9 and ATG16L1, and knockdown of clathrin or AP2 inhibits autophagosome formation (Ravikumar et al., 2010; Popovic and Dikic, 2014). siRNA silencing of CHC or AP2 inhibited intracellular replication and led to reduced CCV size in WT infections, suggesting that CvpA co-opts, rather than inhibits clathrin and AP2 activity (Larson et al., 2013).

The exact mechanism by which CvpA facilitates the accumulation of clathrin at the CCV is currently unknown, but it may stabilize the interaction of clathrin/AP2 at endosomes, retaining them to facilitate fusion at the CCV. It is also currently unknown how CvpA may modulate the induction or progression of autophagy, as well as how it may regulate the endocytosis of molecules beyond Tf. Further, given Cig57 and CvpA both interact with host factors early in the endocytic pathway to facilitate the expansion of the CCV and localization of CHC, it is of interest to determine if there is an interaction between them, or if the activity of one effector is reliant on another. It would also be valuable to observe if a more severe defect develops in the absence of both effectors than in the absence of either one alone.

In addition to CvpA and CvpB, Larson and colleagues also identified CvpC-E, which localized to the CCV and whose absence significantly impaired *C. burnetii* intracellular replication and CCV development in THP-1 cells (Larson et al., 2015). The localization of these proteins to the CCV membrane indicates that they may participate in CCV-autophagosome/endosome fusion events, or the manipulation of the autophagy pathway to promote CCV expansion.

CvpC (CBU1556, Cig50) was also identified by Weber et al. (2013) in a screen for effectors required for intracellular replication, where it was found that CvpC interfered with the host secretory pathway in HEK293T cells. However, when HeLa or J774A.1 cells were infected with a CvpC transposon mutant, no defect in replication was observed (Weber et al., 2013). This may suggest that CvpC activity is specific to human macrophage cells, although further research is needed to confirm this.

CvpD (CBU1818) also appeared in a subsequent screen for *C. burnetii* effector proteins (Lifshitz et al., 2014). CvpD was found to inhibit yeast cell growth (Lifshitz et al., 2014), suggesting it interferes with essential eukaryotic pathways. Interestingly, Wachter et al. (2019) identified a *C. burnetii* sRNA CbsR12, which downregulates CvpD transcripts (Wachter et al., 2019). Absence of CbsR12 has a deleterious effect on both axenic and intracellular replication of *C. burnetii*. Loss of CbsR12 also reduced CCV size, although only early in infection; conversely, its overexpression increases CCV size at both early and late time points (Wachter et al., 2019). This may indicate that the activity of effector proteins such as CvpD may be limited to specific infection phases. However, as with CvpC and CvpE, further information about the biochemical activity of CvpD is currently lacking.

A recent screen by Crabill et al. (2018) also used a transposon library to identify effectors required for efficient CCV biogenesis. In addition to confirming previously identified effectors, seven additional effector mutants were shown to produce small CCVs: CBU0414, CBU0513, CBU0987, CBU1387, CBU1524, CBU1752, and CBU2028, with all except CBU2028 also having impaired intracellular replication. Of these, CBU0987, CBU1387, CBU1524, CBU1752, and CBU2028 were shown to accumulate at the CCV membrane. While the activity of these effectors was not explored, it was observed that in CBU0513 mutants LC3 was not present at the CCV as it was in other mutants and WT *C. burnetii* (Crabill et al., 2018), suggesting a role for this effector in autophagosome-CCV fusion.

Beyond those localizing at the CCV, additional proteins within the *C. burnetii* effector repertoire are likely to interact with the autophagy pathway through other mechanisms. For example, when ectopically expressed the effector CpeB (CBUA0013) colocalizes with LC3B, and CpeL (CBUDA0024) partially colocalized with autophagosomes (Voth et al., 2011; Maturana et al., 2013). Additionally, the protein kinase CstK (CBU0175) was found to localize at vesicles and the CCV and interacts with a homologue of mammalian TBC1D5 in an amoeba model (Martinez et al., 2020). TBC1D5 is a GTPase activating protein for Rab7, which is involved in lysosomal biogenesis, positioning and function, as well as fusion with autophagosomes (Guerra and Bucci, 2016). While the sub-cellular localization of these effectors suggests they may be involved in the manipulation of autophagy, further research is needed to confirm whether they contribute to control of this host pathway and what the functional implications of this are.

## Conclusions

Despite their phylogenetic relationship, *L. pneumophila* and *C. burnetii* have adapted two very different approaches to intracellular replication; however, both manipulate common targets in the autophagy pathway in order to remodel the host cell. The convergence of these targets, despite the unique effector cohorts of *L. pneumophila* and *C. burnetii*, may be a result of their shared ancestry, but can also be seen as an indication of key checkpoints along the autophagic pathway and targets amenable to modifications. By better understanding how *L. pneumophila* and *C. burnetii* effectors interact with the host autophagy pathway, we may also identify approaches common to other intracellular pathogens and uncover mechanisms with which to better understand and control autophagy in the context of human health and disease.

*L. pneumophila* is an intriguing intracellular pathogen whose close association with a broad range of protozoan hosts in the environment has enabled adaptation for survival within human macrophages. Given the known functional redundancy that exists within the large cohort of over 300 effectors translocated by the T4SS, it is not unexpected to discover that multiple effectors are able to influence the host autophagy machinery using a variety of mechanisms (summarised in **Figure 4**). For example, RavZ, which efficiently halts autophagosome maturation through the irreversible cleavage of LC3‑II, is not present in all strains and yet the autophagy machinery is still disrupted (Amer and Swanson, 2005). In this instance, it is easy to speculate that the action of effectors such as *Lp*Spl and Lpg1137 that interfere with autophagosome biogenesis, and the transcription factor Lpg2936 would play a greater role in manipulation of host autophagy. It is also equally plausible that these RavZ-deficient strains possess other, strain-specific, autophagy modulating effectors.

Given the importance of evading autophagy to *Legionella*, the existence of a common cohort of autophagy-related effectors may be considered. However, the recent analysis by Gomez-Valero and colleagues which identified a putative 18,000 effector proteins found only eight were present in all *Legionella* genomes assessed (Gomez-Valero et al., 2019). Further, the ability to infect human cells arose independently multiple times throughout the genus, and no specific set of effectors could be attributed to the ability to infect human cells. Finally, 16 Rab-like proteins from eight different *Legionella* species were found to have been acquired by horizontal gene transfer from hosts (Gomez-Valero et al., 2019). This suggests it is unlikely that a core of autophagy-related effectors exists in the *Legionella* genus. Rather, *Legionella* species will have evolved unique cohorts based on genes acquired from their specific host range.

Since deletion of any of these individual autophagy related effectors has no direct impact on intracellular bacterial replication the coordinated interplay between these effectors are important for *L. pneumophila* avoidance of the host autophagic machinery and subsequent success within host cells. The exact role for LegA9 in enhancing recognition of the LCV for autophagy clearance is yet to be fully elucidated, and no doubt controlling the temporal action of this effector is critical for bacterial survival. Given the redundant nature of many *L. pneumophila* effectors, it would be interesting to remove all known effectors that regulate host autophagy. Whether this would affect intracellular survival and replication or not, and if any changes in pathogenicity were host-specific would provide valuable information about the evolutionary pressures that have led to the retention of these effectors. Subsequent restoration of individual autophagy modulating effectors could then also provide a platform to assess the role of these effectors in isolation. This huge assortment of effectors is almost certain to contain more regulators of autophagy, potentially providing a plethora of novel tools, similar to RavZ, that can be used to explore, uncover and manipulate autophagy at a molecular level.

In contrast, *C. burnetii* relies on a functional autophagy pathway in host cells. However, there is also some evidence that *C. burnetii* down-regulates aspects of autophagy, such as increasing the pH of mature endosomes and decreasing cellular lysosome activity and content (Mulye et al., 2017; Samanta et al., 2019). Indeed, overexpression of TFEB was found to decrease CCV size and bacterial replication during infection (Samanta et al., 2019). Conversely, *C. burnetii* has been found to activate TFEB and the related transcription factor TFE3 *via* inhibition of mTORC1 (Larson et al., 2019; Padmanabhan et al., 2020). Consistent with other studies, this did not result in an increase in autophagy or inhibition of autophagic flux, but TFE3/TFEB were required for efficient CCV development. Macrophage cells with TFE3/TFEB knocked out were curiously able to support higher levels of *C. burnetii* replication despite producing smaller CCVs (Larson et al., 2019), while HeLa cells with dual siRNA silencing of TFEB and TFE3 showed no change in replication, despite still exhibiting a significant decrease in CCV size (Padmanabhan et al., 2020). These results highlight the fine balance *C. burnetii* maintains in modulating the host autophagy system to favour infection and indicates the presence of a range of compensatory and complementary modifications to the host cell. They also highlight the uncertainties surrounding the role of autophagy in the *C. burnetii* lifecycle. While it is often hypothesized that autophagy is induced to supply nutrients to the replicating bacteria, the inconsistent results observed regarding regulators of autophagy suggest this may not be a crucial role. Indeed, the capacity of *C. burnetii* to still replicate in the absence of autophagy argues that this process is not required for bacterial nutrient acquisition. It is possible that other functions of autophagy, such as enhanced/altered vesicle trafficking and fusion are important for reasons beyond the delivery of nutrients. However, the interdependent nature of these two roles has made it difficult to disentangle them experimentally. As our understanding of how *C. burnetii* regulates autophagy develops, it will be worth considering how we can adapt these effectors as potential tools in the treatment of diseases associated with the dysregulation of autophagy, including many neurodegenerative disorders.

## Author Contributions

All authors listed have made a substantial, direct and intellectual contribution to the manuscript and approved it for publication. All authors contributed to the article and approved the submitted version.

## Conflict of Interest

The authors declare that the research was conducted in the absence of any commercial or financial relationships that could be construed as a potential conflict of interest.
